# Rising Above the Flood: Emergency Management and Gerontological Social Work

**DOI:** 10.1093/geroni/igaa057.214

**Published:** 2020-12-16

**Authors:** Susanny Beltran, Paola Luigi, Nancy Kusmaul

**Affiliations:** 1 University of Central FLorida, Orlando, Florida, United States; 2 University of Central Florida, Orlando, Florida, United States; 3 UMBC, Baltimore, Maryland, United States

## Abstract

Older adults, particularly those from low-income communities, are disproportionately negatively affected by natural disasters. As the older adult population grows and natural disasters increase in frequency and intensity, social workers must consider their role in supporting the needs and safety of this population. Social workers practice in varied roles including policy advocate, service broker, and educator, all of which are crucial in disaster management. This systematic review summarizes the literature on the social work profession’s involvement with emergency management with older adults, and identifies gaps. Using the Preferred Reporting Items for Systematic Reviews and Meta-Analyses guidelines, authors searched AgeLine, CINAHL Complete, MEDLINE, PsycINFO, and Social Work Abstracts for peer-reviewed publications between January 1, 2009 and March 1, 2020. Examples of the terms searched include social work*, respon*, prepar*, disaster, crisis, emergency, geriatrics and older adults. The initial searches yielded 298 publications. After removing duplicates and screening articles for relevance based on titles and abstracts, 21 publications were retained for full review. A total of 11 articles met inclusion criteria. The body of literature identified was small. The majority of the publications constituted conceptual papers, textbook reviews, and letters to editors requesting greater emphasis on emergency management. Only three empirical studies were identified. Broadly, the publications discussed: (1) policies and resources; (2) needs; (3) capacity across practice settings; and (4) interventions. Findings reveal an underdeveloped area of social work practice, and highlight opportunities for researchers and practitioners to define gerontological social workers’ role in emergency management and detail best practice guidelines.

